# A high‐throughput transient expression system for rice

**DOI:** 10.1111/pce.13542

**Published:** 2019-04-02

**Authors:** Mike T. Page, Martin A.J. Parry, Elizabete Carmo‐Silva

**Affiliations:** ^1^ Lancaster Environment Centre Lancaster University Lancaster UK

**Keywords:** carboxysome, cellular localisation, confocal microscopy, Golden Gate, high‐throughput, Oryza sativa, protoplasts, Rice, synthetic biology, transient expression

## Abstract

Rice is an important global crop and represents a vital source of calories for many food insecure regions. Efforts to improve this crop by improving yield, nutritional content, stress tolerance, or resilience to climate change are certain to include biotechnological approaches, which rely on the expression of transgenes *in planta*. The throughput and cost of currently available transgenic expression systems is frequently incompatible with modern, high‐throughput molecular cloning methods. Here, we present a protocol for isolating high yields of green rice protoplasts and for PEG‐mediated transformation of isolated protoplasts. Factors affecting transformation efficiency were investigated, and the resulting protocol is fast, cheap, robust, high‐throughput, and does not require specialist equipment. When coupled to a high‐throughput modular cloning system such as Golden Gate, this transient expression system provides a valuable resource to help break the “design‐build‐test” bottleneck by permitting the rapid screening of large numbers of transgenic expression cassettes prior to stable plant transformation. We used this system to rapidly assess the expression level, subcellular localisation, and protein aggregation pattern of nine single‐gene expression cassettes, which represent the essential component parts of the β‐cyanobacterial carboxysome.

## Abstract 2

We provide a protocol for the transient expression of transgenes in rice protoplasts. The method is simple, inexpensive, confers a high efficiency of transformation, and is high‐throughput, thus permitting its use in combination with high‐throughput cloning methods. We demonstrate its utility in assessing the localisation of various cyanobacterial proteins.

## 1 INTRODUCTION

The plant science community is currently faced with many “grand challenges.” These include attaining sustainable global food security, ensuring crops are resilient to climate change, and restoring ecosystems (Huber, 2011). Challenges of this magnitude demand large collaborative research networks (e.g., 2Blades Foundation, http://2blades.org; C4 Rice, www.c4rice.irri.org; ENSA, https://www.ensa.ac.uk/; IWYP, https://iwyp.org/; RIPE, https://ripe.illinois.edu/; ROOTOPOWER, http://www.rootopower.eu) employing state‐of‐the‐art research techniques. Synthetic biology and the molecular cloning systems it has inspired, are sure to play a significant role in providing solutions to these challenges (Cabello, Lodeyro, & Zurbriggen, 2014; Kubis & Bar‐Even, 2019; Piquerez, Harvey, Beynon, & Ntoukakis, 2014). Such cloning systems permit the rapid and efficient assembly of hundreds (by hand) to thousands (with automation) of expression cassettes (De Paoli, Tuskan, & Yang, 2016; Gibson et al., 2009; Patron et al., 2015; Pollak et al., 2019). However, assessing the efficacy of large numbers of expression cassettes via stable transformation remains impractical for many crop species because it is a relatively slow and expensive process, is low‐throughput, confers a low transformation efficiency, and occupies a large volume of growth space (Altpeter et al., 2016). Consequently, there is a practical limit to the number of assembled cassettes that can be tested empirically, thus generating a bottleneck in the so‐called “design‐build‐test” cycle (Sun, Noireaux, & Murray, 2014).

Transient expression platforms have the potential to break this bottleneck (Sainsbury & Lomonossoff, 2014). As the name suggests, these platforms rely on short‐term expression of transgenes in the species of interest, without integration of transgenic cassettes into the host genome. The ideal transient expression platform should be technologically simple, fast, cheap, robust, high‐throughput, and confer high transformation efficiencies, while also providing a means of assessing the efficacy of expression cassettes. Many transient expression platforms for crop species currently rely on the soil bacterium *Agrobacterium tumefaciens* (also known as *Rhizobium radiobacter*) to deliver expression cassettes located on T‐DNA into plant nuclei (Andrieu et al., 2012; Bhaskar, Venkateshwaran, Wu, Ané, & Jiang, 2009; Panwar, McCallum, & Bakkeren, 2013). While these methods can confer suitable levels of transgene expression *in planta*, they are low‐throughput and often require tissue from mature plants, increasing the growth space required and the time taken to acquire data. Moreover, infection by A. tumefaciens can induce widespread gene expression changes in the host, which may influence the dynamics of transgene expression (Ditt et al., 2006). Another approach is the bombardment of plant tissue with DNA‐carrying microprojectiles (Kirienko, Luo, & Sylvester, 2012; Ueki, Magori, Lacroix, & Citovsky, 2013), but such procedures require specialist equipment, are not particularly high‐throughput and can be expensive due to the cost of DNA microcarriers. Transformation of plant protoplasts has also been widely used and confers many advantages over other methods. It does not require specialist equipment, allows high‐throughput screening, and is relatively cheap, fast, and robust. In addition, protoplasts are routinely isolated from young tissue (7–10‐day‐old seedlings), reducing growth space and time.

Here, we describe a method for the isolation and transformation of protoplasts from the widely researched rice variety Nipponbare (Oryza sativa ssp. japonica). The method has been developed based on previous protoplast transformation protocols (Shan, Wang, Li, & Gao, 2014; Shen et al., 2014; Xie & Yang, 2013; Yoo, Cho, & Sheen, 2007; Zhang et al., 2011) and importantly made technologically simpler to facilitate its widespread uptake. Coupled with an efficient molecular cloning platform such as Golden Gate, this method enabled high‐throughput screening and optimisation of transgenic cassettes prior to stable plant transformation.

## 2 MATERIALS AND METHODS

### 2.1 Growth of plant material

**Figure 1:**
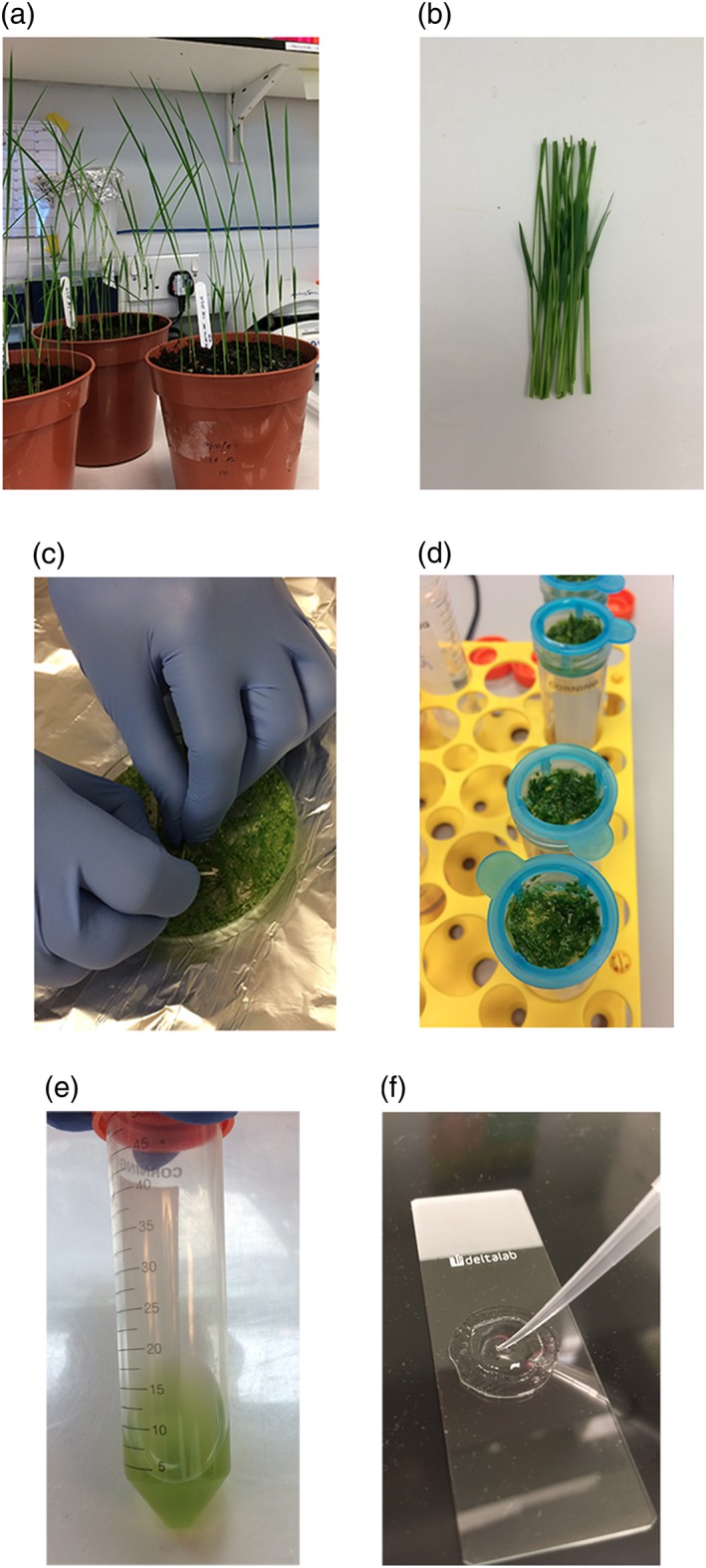
Photographs of various steps in the protocol. Rice seedlings 7 days after germination (a), approximately 100 mm of rice stem and sheath tissue (b), cutting tissue into mannitol to initiate plasmolysis (c), filtration of digested tissue through 40 μm mesh (d), protoplast suspension after isolation (e), and mounting transformed protoplasts onto slides in a well constructed from observation gel (f). Scale bars represent 45 mm (a), 20 mm (b), 25 mm (c), 15 mm (d), 15 mm (e), 10 mm (f)

Seventy‐five O. sativa ssp. japonica Nipponbare seeds were sown into wet compost (Rothamsted mix, Petersfield Growing Mediums, Leicester, UK) at a depth of approximately 15 mm and covered loosely with soil. If seeds were freshly harvested, dormancy was broken by incubating at 60°C for 3 days. The seeds were transferred to a growth chamber with the following environmental settings: 12 hr light at ~500 μmol photons m^−2^ s^−1^, 30°C, 60% relative humidity and 12 hr dark at 26°C, 60% humidity. Plants were allowed to grow for 7 days after germination (Figure 1a).

### 2.2 Assembly and preparation of plasmid DNA (pDNA)

All plasmids used in this manuscript were assembled with Golden Gate cloning. A 2:1 molar ratio of insert:acceptor was used for all reactions in a total volume of 15 μL. These reactions employed the type IIS restriction enzyme *Bsa*I and included ATP at a final concentration of 1 mM. Expression of cyanobacterial genes was driven by a combination of well‐characterised promoters that were domesticated and synthesized *de novo* (*pOsAct1*, *pZmUbi1*, *pOsRbcs2*, *pZmPEPC*) and promoters from the MoClo Plant Parts kit (Addgene kit # 1000000047; Engler et al., 2014). The turbo GFP (*tGFP*) and *DsRed* sequences were also obtained from the MoClo Plant Parts kit. Cyanobacterial gene sequences, yellow fluorescent protein (YFP), and the stromal transit peptide sequence from Arabidopsis thaliana *RecA* (Cerutti, Osman, Grandoni, & Jagendorf, 1992) were domesticated, codon‐optimised for expression in rice and synthesized *de novo* (Genscript, Piscataway, USA). Codon‐optimised versions of *CcmK2*, *CcmL*, and *CcmO* were a kind donation from Prof Maureen Hanson (Cornell University, USA) and were domesticated where required. The chloroplast envelope transit peptide (ceTP) was created by combining the RecA transit peptide with a cyanobacterial protein known to localise to the cyanobacterial cell membrane. Reactions were subject to 30 cycles of digestion (37°C, 2 min) and ligation (16°C, 3 min), followed by a longer final digestion step (37°C, 5 min) and an incubation at 80°C for 10 min. ElectroMAX™ DH10B™ Escherichia coli cells (Thermo Scientific, Waltham, USA) were transformed with 0.5 μL reaction mix and selected on the appropriate antibiotic.

A single E. coli colony was used to inoculate 5 ml liquid LB (10 g L^−1^ tryptone, 5 g L^−1^ bacto‐yeast, pH 7.0) containing the appropriate antibiotic. Cultures were shaken at 37°C and 175 rpm for 12–16 hr. Bacterial cells were pelleted by centrifuging cultures at 3,000 × g for 10 min. pDNA was extracted from the cells using the GeneJET Plasmid Miniprep kit (Thermo Scientific) and eluted in 50 μL ddH_2_O. Concentration and quality were determined using spectrophotometry, and sequences of expression cassettes were verified using Sanger sequencing (primer sequences are given in Supplementary Table S1). Plasmid maps are given in Supplementary Figure S1.

### 2.3 Isolation of protoplasts

Sixty seedlings were harvested by cutting the stem at the base of the plant at soil level. Approximately 100 mm of stem and sheath tissue was retained (Figure 1b) and briefly rinsed with water to remove any adhered compost. The plant tissue was cut into ~1 mm slices using a sharp blade, directly into 40 ml 0.6 M mannitol inside a Petri dish (Figure 1c). The tissue was incubated in the dark for 15 min at room temperature (RT) to initiate plasmolysis. After draining off the mannitol solution, the tissue was transferred to a conical flask containing 30 ml enzyme solution (20 mM 2‐(N‐morpholino)‐ethanesulfonic acid (MSE) pH 5.7, 1.5% *w*/*v* cellulase RS, 0.75% *w*/*v* macerozyme R10, 0.6 M mannitol, 10 mM KCl, 10 mM CaCl_2_, 0.1% *w*/*v* bovine serum albumin (BSA)) and incubated in the dark for 4 hr at RT with gentle shaking to allow digestion of cell wall material; 30 ml W5 solution (2 mM MES pH 5.7, 154 mM NaCl, 125 mM CaCl_2_, 5 mM KCl) was added and the flask shaken gently by hand for 20 s to terminate digestion. Samples were filtered through 40 μm mesh by gravity to release protoplasts, and the retained tissue rinsed with an additional 60 ml W5 solution (Figure 1d). Protoplasts were pelleted by centrifuging all flow‐through from filtering and washing (120 ml total) at 250 × g for 3 min at RT. The supernatant was carefully decanted, and the pellets washed by gentle resuspension in 10 ml W5 buffer, then centrifugation at 250 × g for 3 min at RT. The supernatant was again carefully decanted, and pellets were resuspended in 2 ml MMG solution (4 mM MES pH 5.7, 0.4 M mannitol, 15 mM MgCl_2_) (Figure 1e). A detailed protocol is available as Supplementary Protocol S1 and Supplementary Video S1.

### 2.4 Transformation of protoplasts

5 μg pDNA (in a volume of 10 μL, diluted with ddH_2_O) and 60 μL protoplast suspension were combined in a 1.5 mL microcentrifuge tube; a negative control sample (60 μL protoplasts, 10 μL ddH_2_O, no pDNA) was included for each round of transformations. 70 μL poly(ethylene glycol) (PEG) solution (0.2 M mannitol, 40% *w*/*v* PEG 4,000, 0.1 M CaCl_2_) was added, and the samples mixed gently by inversion. Tubes were incubated in the dark at RT for 25 min to allow transformation to occur. The transformation process was terminated by addition of 280 μL W5 solution followed by gentle mixing. Samples were centrifuged at 250 × g for 3 min at RT, and the supernatant carefully aspirated. Pellets were resuspended in 500 μL WI solution (4 mM MES pH 5.7, 0.5 M mannitol, 20 mM KCl) and dispensed in 125 μL aliquots into a 96‐well microplate. Transformed protoplasts were incubated at RT on the lab bench for 16 hr, to allow transgenic protein(s) to accumulate. For further details, see Supplementary Protocol S1 and Supplementary Video S1.

### 2.5 Confocal imaging of transformed protoplasts

Protoplasts were resuspended with gentle pipetting and a small drop placed onto a microscope slide inside a well constructed from observation gel (Blades Biological, Edenbridge, UK; Figure 1f). A cover slip was placed on top of the sample, and gentle pressure applied to seal the cover slip onto the observation gel.

All confocal images were acquired with a Zeiss LSM 880 microscope. Excitation lasers for the confocal microscope were switched on and fully warmed up for at least 30 min prior to imaging. Samples were imaged in a random order for each independent experiment to account for any potential effect of the age of the isolated protoplasts. Fluorescent signals were visualised using the following settings: tGFP, excitation = 488 nm, emission = 495–535 nm; YFP, excitation = 488 nm, emission = 505–545; DsRed, excitation = 563 nm, emission = 570–610 nm; chlorophyll autofluorescence: excitation = 488 nm, emission = 670–705 nm.

### 2.6 Statistical analysis

To determine statistical significance of differences between sample means, a one‐way ANOVA was performed, followed by a Tukey's post hoc test for multiple pairwise comparisons (*p* < 0.05). Box‐whisker plots were generated using the ggplot2 package (Wickham, 2016) within RStudio (RStudio Team, 2015).

## 3 RESULTS

### 3.1 Protoplast isolation and transformation efficiency

**Figure 2:**
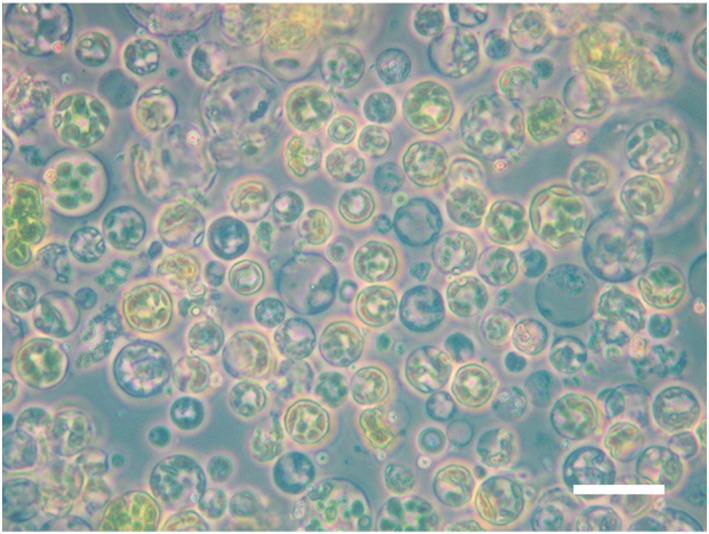
Isolated protoplasts imaged using light microscopy. Isolated protoplasts were imaged with a Zeiss Axioskop 40 microscope under 40x magnification. Scale bar represents 40 μm

The method outlined here enabled isolation of a high yield of intact rice leaf protoplasts. To demonstrate this, a sample of isolated protoplasts was examined using light microscopy. A large proportion of the protoplasts imaged were spherical, indicating they remained intact after the isolation process (Figure 2).

**Figure 3:**
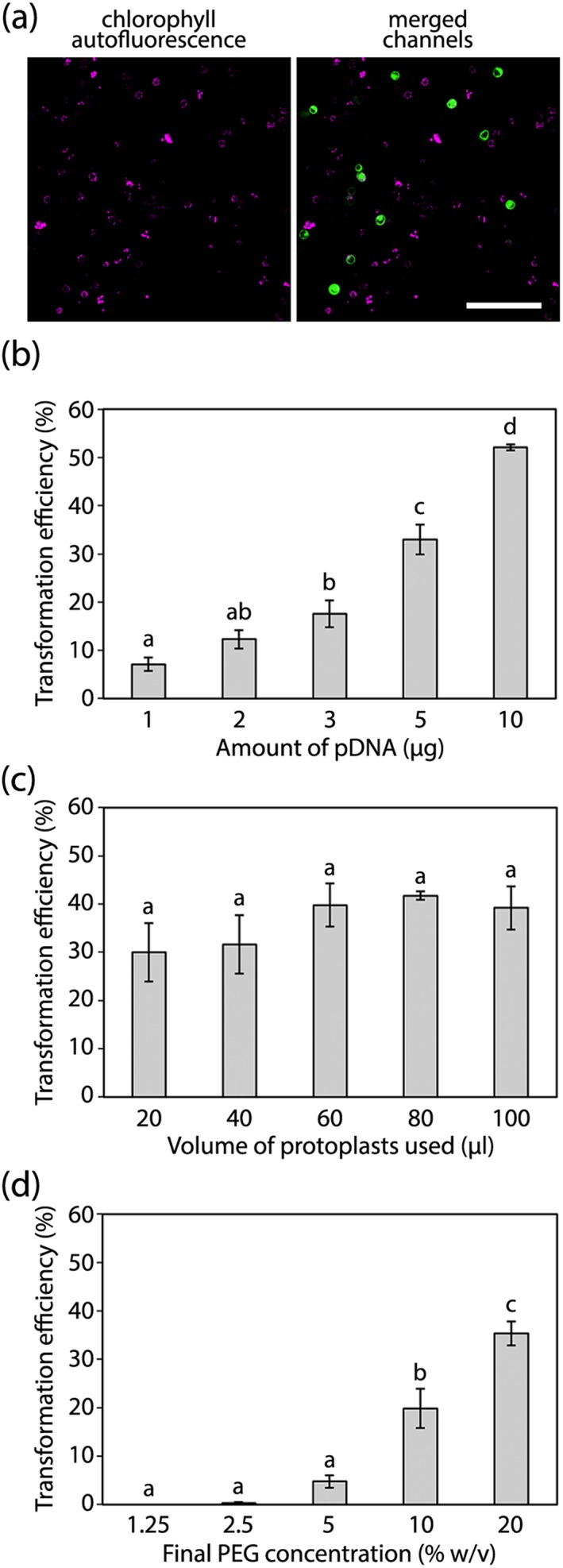
Factors affecting the transformation efficiency of isolated protoplasts. Representative images of the type of data acquired for this assay (a), showing chlorophyll autofluorescence and its overlay with tGFP. Scale bar represents 100 μm. Assessment of protoplast transformation efficiency under varying amounts of plasmid DNA (pDNA; b), volumes of isolated protoplasts (c), or concentrations of PEG (d). These tests were conducted on three biological replicates (protoplasts isolated from three independent batches of rice plants grown over a period of 4 months). In (b) the volume of isolated protoplasts added was fixed at 60 μL, and the final PEG concentration was fixed at 20%. In (c) the amount of pDNA added was fixed at 5 μg, and the final PEG concentration was fixed at 20%. In (d) the amount of pDNA added was fixed at 5 μg, and the volume of isolated protoplasts added was fixed at 60 μL. For each treatment within each biological replicate, six representative and nonoverlapping images (consisting of between 125 and 350 protoplasts) were acquired for analysis. Bars represent the mean of the three biological replicates, error bars represent ±1 × SEM. For the different treatments, lowercase letters denote significant differences between sample means (p < 0.05)

To determine the factors affecting the transformation efficiency of isolated protoplasts, a series of tests was conducted in which the amount of pDNA, the volume of protoplasts, or the concentration of PEG was varied. In each case, protoplasts were transformed with an expression cassette conferring constitutive expression of a fluorescent marker, in this case *tGFP*, in the cytosol. The tGFP protein matures rapidly and is highly soluble (Evdokimov et al., 2006), and the specific variant used here has been codon‐optimised for expression in plants (Engler et al., 2014). Using confocal microscopy, the total number of protoplasts in each image was determined using chlorophyll autofluorescence, whereas the number of transformed protoplasts was determined by the presence of cytosolic tGFP signal (Figure 3a), thus allowing a calculation of the transformation efficiency. The separation of the two signals, both spatially and based on the differing emission spectra of chlorophyll and tGFP, minimised the possibility of identifying false positives. In addition, the expression of *tGFP* was driven by the very strong Ubiquitin1 promoter from Zea mays (*pZmUbi1*), reducing the possibility of detecting background signal.

Protoplast transformation efficiency was positively correlated with the amount of pDNA added to the transformation mix, with 10 μg of pDNA conferring a mean efficiency of over 50% (Figure 3b). However, we recommend adding 5 μg of pDNA per transformation as this would allow a single pDNA miniprep isolation to provide sufficient pDNA for several transformations (assuming a reasonable plasmid copy number), whereas addition of 10 μg pDNA per transformation would require a larger scale pDNA isolation method. During imaging of this experiment, it was consistently observed that addition of higher amounts of pDNA generally led to higher tGFP signals, whereas the addition of lower amounts of pDNA generally led to weaker tGFP signals. This was quantified by imaging the first 35 protoplasts observed in each sample using consistent microscope settings (laser power and gain) and assessing the tGFP signal intensity. These data demonstrated that pDNA amount was indeed positively correlated with the median and maximum tGFP signal (Supplementary Figure S2). It is assumed that this phenomenon occurs as transforming protoplasts with higher amounts of pDNA will increase the likelihood that protoplasts take up a high number of copies of the same plasmid and thus exhibit a higher tGFP signal.

There was no significant difference in transformation efficiency when protoplast volume was varied (Figure 3c). This indicates that the use of smaller volumes or protoplast preparations of low yield, will still enable successful transformation. We recommend adding a volume of 60 μL of protoplasts per transformation, as lower volumes lead to relatively dilute samples for downstream analysis. Like pDNA amount, the final concentration of PEG in the transformation mix correlated positively with transformation efficiency (Figure 3d). The highest concentration tested here (20% PEG) is recommended, as concentrations above 20% make the PEG solution difficult to pipette accurately.

### 3.2 A demonstration of the protocol to assess multiple single‐gene expression cassettes

**Figure 4:**
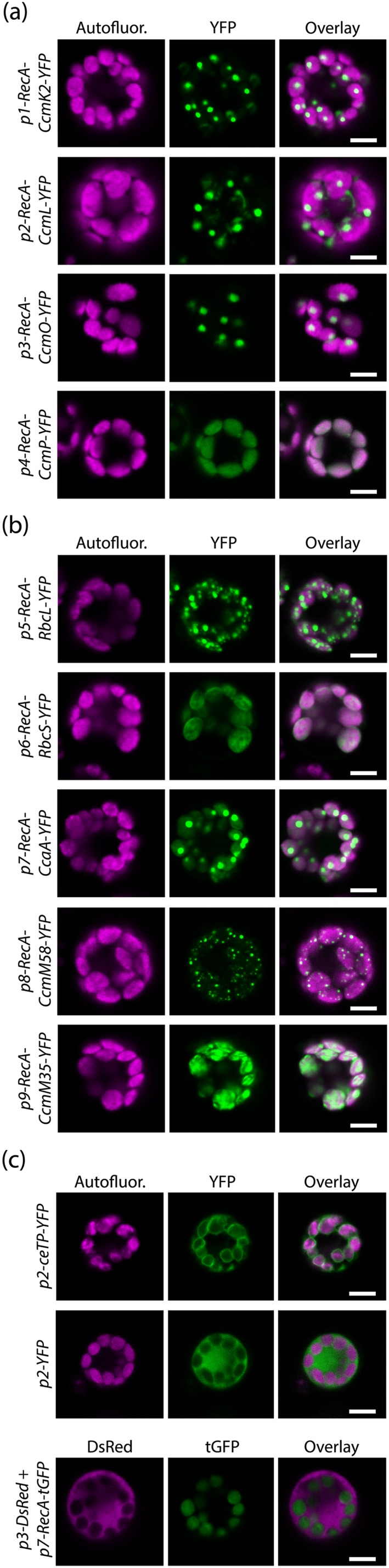
Localisation of cyanobacterial proteins targeted to rice chloroplasts. Confocal microscopy images showing chlorophyll autofluorescence (“Autofluor.”), yellow fluorescent protein (YFP) signal, and the overlay of these two channels for carboxysome shell (a) and internal (b) YFP fusion proteins. “p1”–“p9” denote the different promoters used to drive expression of the various cyanobacterial transgenes. Additional examples of subcellular localisation of fluorescent proteins (c): to the chloroplast envelope using a chloroplast envelope transit peptide (ceTP), to the cytosol in the absence of a transit peptide, and the simultaneous localisation of two fluorescent proteins (DsRed and tGFP) to different compartments. All images are representative for each cassette. The same magnification (400×) was used to acquire all images, scale bars represent 10 μm

The value of this protocol was demonstrated through the independent expression of β‐cyanobacterial carboxysome genes in rice chloroplasts. The carboxysome microcompartment represents the hub of the carbon dioxide concentrating mechanism (CCM) in cyanobacteria and is composed of a proteinaceous shell containing rubisco, carbonic anhydrase, and other structural proteins. Installation of the carboxysome into crop plants lacking a CCM represents a viable strategy to sustainably increase crop yields (Hanson, Lin, Carmo‐Silva, & Parry, 2016; Price et al., 2013). Nine single‐gene expression cassettes each possessing a C‐terminal fusion to a fluorescent protein, in this case YFP, were used to independently transform isolated rice protoplasts. Confocal imaging revealed that the YFP signal was at a level suitable for signal detection above background levels for the shell (Figure 4a) and internal (Figure 4b) protein fusions. Further, it highlighted differences in transgene expression level between cassettes, an expected result almost certainly conferred by the use of different promoters in each cassette. This provides valuable information in determining the optimum promoter‐gene combinations to achieve the desired stoichiometry of subunits in order to assemble functional carboxysomes.

Transient expression of β‐cyanobacterial carboxysome genes using protoplasts also confirmed that all nine proteins were successfully targeted to rice chloroplasts because YFP signal colocalised with chlorophyll autofluorescence in each case. Stromal targeting was conferred through incorporation of the transit peptide from the *RecA* gene of A. thaliana (Cerutti et al., 1992) immediately upstream of each cyanobacterial gene sequence. The data further demonstrated that there were differences in the distribution of the transgenic proteins in the chloroplast stroma. The majority of carboxysome protein‐YFP fusions conferred discrete fluorescent foci, either as fewer larger foci (CcmK2‐YFP, CcmL‐YFP, CcmO‐YFP, CcaA‐YFP), or a larger number of smaller foci (RbcL‐YFP, CcmM58‐YFP), suggesting these protein fusions are able to self‐assemble. On the contrary, CcmP‐YFP and RbcS‐YFP appeared to be diffuse in the chloroplast stroma, whereas CcmM35‐YFP gave a pattern somewhat between diffuse and defined foci. The degree of protein self‐assembly was not linked to expression level conferred by the different promoters but instead closely aligned with the known functions of the proteins in the cyanobacterial carboxysome (Price et al., 2013). In addition, the assembly pattern of the proteins shown in Figure 4a,b was observed consistently across each sample. For example, when observing the first 100 protoplasts in each sample for *p5*‐*RecA*‐*RbcL*‐*YFP* and *p1*‐*RecA*‐*CcmK2*‐*YFP*, the pattern of assembly shown in Figure 4a,b was observed in 98 and 99 protoplasts, respectively. The ability to distinguish between assembly states will be important in determining the efficacy of cassettes when individual carboxysome genes are expressed in combination.

Having demonstrated successful targeting of carboxysome proteins to chloroplasts, we tested whether we could direct YFP to other subcellular locations within protoplasts. When YFP was fused to a transit peptide conferring chloroplast envelope localisation (ceTP), YFP signal was restricted to the area immediately surrounding chlorophyll autofluorescence signal (Figure 4c), indicating successful localisation to chloroplast envelopes. In the absence of a transit peptide, the expected pattern of cytosolic localisation was observed. We also tested whether two fluorescent proteins could be directed to different subcellular compartments within the same protoplast. A single plasmid was used to express DsRed without a transit peptide and tGFP with a chloroplast transit peptide, and the resulting signal pattern showed successful localisation of DsRed in the cytosol and tGFP within chloroplasts (Figure 4c). These data confirm that the transient transformation system described here is ideally suited for subcellular localisation studies.

## 4 DISCUSSION

The transient expression system described here has been developed for rice protoplasts to enable rapid screening of large numbers of transgenic expression cassettes. Rice is responsible for providing approximately one fifth of the global daily calorie intake and represents a staple carbohydrate in many food insecure regions (Ray, Mueller, West, & Foley, 2013). There are currently many large collaborative research projects (e.g., C4 Rice, www.c4rice.irri.org; RIPE, www.ripe.illinois.edu) attempting to enhance rice yields that would benefit greatly from a high‐throughput transient expression platform such as this for screening transgenic cassettes.

The protocol described here is robust, provided that a few critical steps are followed carefully. The age of seedlings used for protoplast isolation should be between 7 and 10 days, although we have used 14 days old seedlings with little reduction in protoplast yield or transformation efficiency. Beyond 14 days of growth, the protoplast yield declines sharply, presumably due to the strengthening of cell walls as cell expansion slows. To avoid rupturing protoplasts, the centrifugation speed should not exceed 250 × g, and any mixing should be done gently by hand rather than vortex.

Previous protoplast isolation and transformation protocols have emphasized the need for vacuum infiltration of plasmolysed leaf tissue with enzyme solution prior to the 4 hr incubation to obtain high protoplast yields (Shan et al., 2014; Yoo et al., 2007), although we have obtained excellent yields of healthy protoplasts without this treatment. We also found it unnecessary to check for protoplast release after digestion, a step that is included in some protocols (Yoo et al., 2007). We also removed the cell counting step, which assesses the density of isolated protoplasts prior to transformation (e.g., with a haemocytometer). Instead, we found that the use of a consistent quantity of rice seedling tissue was sufficient to confer a consistent transformation efficiency. In addition, we achieved a transformation efficiency of 30% or above for all volumes of protoplasts tested (Figure 3b), indicating that low protoplast yields would be compatible with this protocol (although they will reduce the number of protoplasts available for analysis). Using confocal imaging, we were able to acquire data 16–20 hr after protoplast transformation because the confocal signal was consistent and robust at this time. This, in conjunction with the omission of the steps outlined above, dramatically shortened the time taken to acquire data compared with previous protocols (Shan et al., 2014; Shen et al., 2014; Xie & Yang, 2013; Yoo et al., 2007) and therefore increased the throughput of this approach accordingly. This enables the coupling of this protocol to a high‐throughput cloning platform to test the efficacy of large numbers of expression cassettes very quickly. We have also shown that results can be obtained using considerably less pDNA than previously indicated (Shan et al., 2014; Shen et al., 2014; Xie & Yang, 2013; Yoo et al., 2007; Zhang et al., 2011), with the consequence that simple small‐scale pDNA isolations (minipreps) are sufficient to allow multiple independent replicates of protoplast transformation. By omitting the vacuum infiltration and cell counting steps, this protocol is readily adoptable by any laboratory with basic equipment, facilitating its widespread uptake.

An important consideration when using this method is the manner in which the efficacy of each transgenic cassette will be determined. To fully exploit the speed and high‐throughput nature of this protocol, a simple visual assay (e.g., a fluorescent reporter assayed via confocal microscopy) is recommended. If transformed protoplasts are to be imaged via confocal microscopy, the optimum time for imaging is 16–24 hr after completing Section 4 of the protocol. After this window, the fluorescent signal reduces rapidly, presumably due to the deteriorating health of the protoplasts.

This method confers several advantages over other transient expression systems. First, it does not require A. tumefaciens to deliver T‐DNA into plant cells, instead relying on PEG‐mediated uptake of pDNA (Hayashimoto, Li, & Murai, 1990). This eliminates the need for an additional bacterial transformation step (pDNA into A. tumefaciens), reducing the time to acquire data. It also simplifies the optimisation of this method for other varieties or species, as compatibilities between A. tumefaciens strains, and plant species/varieties need not be addressed. Second, this method is high‐throughput and rapid, permitting the screening of over 30 (single or multigene) expression cassettes for every batch of isolated protoplasts, with data acquired within 36 hr of commencing protoplast isolation (if using confocal microscopy to determine transgene cassette efficacy). The entire schedule of molecular cloning, transient transformation, and data acquisition and analysis for the nine expression cassettes in Figure 4 was completed within 2 weeks. Third, the method is cheap and technologically simple. Isolation and transformation of a single batch of protoplasts cost approximately £40 based on the consumables used here (£1.20 per cassette if 60 μL protoplasts are used per transformation), did not use any specialist equipment and used very little plant growth space. This will facilitate adoption of this method by researchers, including those in less economically developed countries. Finally, confocal imaging is considerably simpler in isolated protoplasts than intact plant tissues. This is due to the absence of a waxy cuticle layer in isolated protoplasts, as well as the elimination of virtually all out‐of‐focus fluorescence from above or below the focal plane.

Given the advantages laid out above, in addition to the localisation studies performed here, this platform could be used to answer a wide range of research questions. Because transgenic mRNAs will be processed by the native rice translation machinery in the presence of any required chaperones, rice proteins are likely to be folded correctly and will interact in an environment representative of intact leaf tissue. As such, this platform is suitable for protein expression prior to *in vitro* analysis or for screening protein–protein interactions *in* *vivo* using bimolecular fluorescence complementation (Kerppola, 2006). It could also be used in functional studies, for example, by comparing the relative strengths of different promoters, through the rapid screening of promoter deletion libraries, or comparing protein fusion cassettes (e.g., different tags/linkers, N‐ *vs*. C‐terminal fusions). Additionally, the platform presented here could be used to screen the efficacy of gRNAs in CRISPR/Cas9 genome editing or even assess more recent advancements in CRISPR/Cas9 technology such as DNA methylation editing or base editing (Gaudelli et al., 2017; Liu et al., 2016).

## Supporting information

Data S1. Supplementary Protocol S1. A detailed protocol for rice protoplast isolation and transformation, including reagents and materials required.

Video S1. A time‐lapse video to accompany Supplementary Protocol S1, with numbered steps matching the steps in the written protocol.

Table S1. Sequencing primers used to verify correct assembly of plasmids. Primer binding positions can be seen on the plasmid maps in Supplementary Figure S1.
Supplementary Figure S1(a). Map of Plasmid 1. This plasmid was used for the determination of transformation efficiency when altering pDNA amount, protoplast volume, and final PEG concentration (data in Figure 3b‐d). Primer sequences are given in Supplementary Table S1.
Supplementary Figure S1(b). Map of Plasmid 2. This plasmid was used for localisation studies of RecA‐CcmK2‐YFP (data in Figure 4a). Primer sequences are given in Supplementary Table S1.
Supplementary Figure S1(c). Map of Plasmid 3. This plasmid was used for localisation studies of RecA‐CcmL‐YFP (data in Figure 4a). Primer sequences are given in Supplementary Table S1.
Supplementary Figure S1(d). Map of Plasmid 4. This plasmid was used for localisation studies of RecA‐CcmO‐YFP (data in Figure 4a). Primer sequences are given in Supplementary Table S1.
Supplementary Figure S1(e). Map of Plasmid 5. This plasmid was used for localisation studies of RecA‐CcmP‐YFP (data in Figure 4a). Primer sequences are given in Supplementary Table S1.
Supplementary Figure S1(f). Map of Plasmid 6. This plasmid was used for localisation studies of RecA‐RbcL‐YFP (data in Figure 4b). Primer sequences are given in Supplementary Table S1.
Supplementary Figure S1(g). Map of Plasmid 7. This plasmid was used for localisation studies of RecA‐RbcS‐YFP (data in Figure 4b). Primer sequences are given in Supplementary Table S1.
Supplementary Figure S1(h). Map of Plasmid 8. This plasmid was used for localisation studies of RecA‐CcaA‐YFP (data in Figure 4b). Primer sequences are given in Supplementary Table S1.
Supplementary Figure S1(i). Map of Plasmid 9. This plasmid was used for localisation studies of RecA‐CcmM58‐YFP (data in Figure 4b). Primer sequences are given in Supplementary Table S1.
Supplementary Figure S1(j). Map of Plasmid 10. This plasmid was used for localisation studies of RecA‐CcmM35‐YFP (data in Figure 4b). Primer sequences are given in Supplementary Table S1.
Supplementary Figure S1(k). Map of Plasmid 11. This plasmid was used determine localisation of a fluorescent protein (YFP) directed by a chloroplast envelope transit peptide (ceTP, data in Figure 4c). Primer sequences are given in Supplementary Table S1.
Supplementary Figure S1(l). Map of Plasmid 12. This plasmid was used determine localisation of a fluorescent protein (YFP) in the absence of a transit peptide (data in Figure 4c). Primer sequences are given in Supplementary Table S1.
Supplementary Figure S1(m). Map of Plasmid 13. This plasmid was used determine localisation of two fluorescent proteins: DsRed in the absence of a transit peptide, and tGFP directed by a chloroplast stroma transit peptide (RecA, data in Figure 4c). Primer sequences are given in Supplementary Table S1.
Supplementary Figure S2. The effect of pDNA amount on tGFP signal intensity. Box‐whisker plots to show the distribution of signal intensities when different amounts of plasmid DNA (pDNA) are used to transform protoplasts. The boxes represent the lower quartile, median, and upper quartile. Whiskers extend to the minimum and maximum data values for each sample (excluding outliers). Consistent confocal microscope settings (laser power, gain) were used to image all samples. All individual data points are shown in blue (n = 35).
