# Supplementary material for: A high‐throughput transient expression system for rice
Source: Plant Cell Environ. 2019 Apr 2;42(7):2057–64. doi: 10.1111/pce.13542 (PMC6618034; doi:10.1111/pce.13542)
Supplement: Supplementary file 1 — Data S1. Supplementary Protocol S1. A detailed protocol for rice protoplast isolation and transformation, including reagents and materials required. [file PCE-42-2057-s001.pdf]

A high-throughput transient expression system for rice -- WORKING PROTOCOL

A visual representation of this protocol can be seen in accompanying Supplementary Video S1

Materials, reagents & equipment required

Reagents
Yeast extract (Neogen Europe Ltd., MC001)
Tryptone (Neogen Europe Ltd., MC005)
D-mannitol (Sigma-Aldrich, M4125)
MES monohydrate (Melford, B2002)
Potassium hydroxide (Sigma-Aldrich, 484016)
Cellulase onozuka RS (Duchefa, C8003)
Macerozyme R-10 (Duchefa, M8002)
Bovine serum albumin (BSA, Melford, A1302)
Calcium chloride (Fisher Scientific, C/1400/53)
Potassium chloride (Sigma-Aldrich, P9333)
Sodium chloride (Sigma-Aldrich, 31434)
Magnesium chloride (Sigma-Aldrich, M2393)

Materials & Equipment
Oryza sativa ssp. *japonica* Nipponbare seeds
Compost (Petersfield Mediums, Rothamsted mix)
Syringe filters 0.2 μm (Cole-Parmer, OU-02915-08)
Cell strainer 40 μm nylon (Falcon, 352340)
Centrifuge (VWR, Mega Star 600R)
Light microscope (Zeiss, Axioskop 40)
Poly(ethylene glycol)-4000 (Sigma-Aldrich, 81240)
96-well microplates (Thermo Scientific, 442404)
Observation gel (Blades Biological, CBA047)
Confocal microscope (Zeiss, LSM 880)
GeneJET miniprep kit (Thermo Scientific, K0503)

Reagent Preparation

0.6 M mannitol: Dissolve mannitol in ddH_2O and filter sterilize through a 0.2 μm filter.

0.5 M MES, pH 5.7: Dissolve MES in ddH_2O (may need heat to fully dissolve MES). Adjust to pH 5.7 with potassium hydroxide. Filter sterilize through a 0.2 μm filter and store at 4 °C.

Enzyme Solution (1.5 % w/v cellulase-RS, 0.75 % w/v macerozyme-R10, 0.6 M mannitol, 20 mM MES pH 5.7, 10 mM KCl, 10 mM $CaCl_2$, 0.1 % w/v BSA): PREPARE FRESH. Combine the cellulase RS, macerozyme R-10, mannitol, MES, and KCl in a conical flask. Add 25 ml ddH_2O and warm the solution to 55 °C for 15 min. Cool the solution to room temperature. Add the $CaCl_2$ and BSA, then adjust volume to 30 ml with ddH_2O. Ensure the BSA has dissolved before filter sterilizing through a 0.2 μm filter.

W5 Solution (2 mM MES pH 5.7, 154 mM NaCl, 125 mM $CaCl_2$, 5mM KCl): Combine the MES, NaCl, $CaCl_2$, KCl, and dissolve in ddH_2O. Sterilize by autoclaving.

MMG Solution (4 mM MES pH 5.7, 0.4 M mannitol, 15 mM $MgCl_2$): Combine the MES, mannitol and $MgCl_2$, and dissolve in ddH_2O. Filter sterilize through a 0.2 μm filter.

PEG Solution (40 % w/v PEG-4000, 0.2 M mannitol, 0.1 M $CaCl_2$): PREPARE FRESH. Combine the PEG-4000, mannitol and $CaCl_2$, and dissolve in ddH_2O. *NOTE – the PEG-4000 will take at least 2 h to dissolve, so prepare this at the end of step 3.3.*

WI Solution (0.5 M mannitol, 20 mM KCl, 4 mM MES pH 5.7): Combine the mannitol and MES, and dissolve in ddH_2O. Filter sterilize through a 0.2 μm filter.

1. Growth of plant material

1.1 Sow 75 *Oryza sativa* japonica Nipponbare seeds into wet compost at a depth of approximately 15 mm. Cover loosely with soil. *NOTE – if seeds have been freshly harvested, break dormancy by incubating at 60 °C for 3 d.*

1.2 Transfer seeds to a growth chamber or glasshouse with the following environmental settings: 12 h light (approximately 500 µmol m^{-2} s^{-1}, 30 °C, 60 % relative humidity), 12 h dark (26 °C, 60 % humidity).

1.3 Allow plants to grow for 7 d after germination. *NOTE – Growth for less time will result in lower protoplast yields due to the reduction in starting material. Growth for longer than 14 d will also reduce protoplast yields as the cell walls strengthen and become harder to digest.*

2. Preparation of plasmid DNA (pDNA)

2.1 Pick a single *Escherichia coli* colony (previously transformed with a plasmid of interest) into 5 mL liquid LB (10 g L^{-1} tryptone, 5 g L^{-1} bacto-yeast, pH 7.0) with the appropriate antibiotic.

2.2 Shake at 37 °C and 175 rpm for 12 – 16 h. Pellet the bacterial cells by centrifuging the cultures at 3,000 x g for 10 min.

2.3 Extract the pDNA from the cells using a commercially available miniprep kit. Elute pDNA in 50 µL ddH_2O. *NOTE – we used the GeneJET Plasmid Miniprep Kit (Thermo Scientific, Waltham, USA).*

2.4 Determine the concentration and quality (e.g. by running on an agarose gel or using spectrophotometry). Verify the sequence of the expression cassette(s) using Sanger sequencing. *NOTE – transformation of protoplasts in step 4 requires a minimum pDNA concentration of 500 ng/µl. This is achievable for high or medium copy plasmids using a miniprep kit. For low copy plasmids, perform 3 minipreps for each plasmid and combine at the elution step.*

3. Isolation of protoplasts

3.1 Harvest 60 rice seedlings by cutting the stem at the base of the plant (at soil level). Retain only the stem and sheath tissue (approximately 100 mm). Rinse the seedlings briefly with water to remove any adhered compost. *NOTE – depending on the germination rate of the seeds, a surplus of seedlings should be present. Choose the most established seedlings.*

3.2 Pour 40 mL 0.6 M mannitol into a Petri dish. In the mannitol solution, cut the plant tissue into 1 mm slices using a sharp blade. Incubate in the dark for 15 min at room temperature (RT) to initiate plasmolysis. *NOTE – ensure the plant tissue is submersed in the mannitol solution during cutting.*

3.3 Drain off the mannitol solution, then transfer the plant tissue to 30 mL Enzyme Solution in a conical flask. Incubate in the dark for 4 h at RT with gentle shaking to allow digestion of cell wall material. *NOTE – longer incubations (> 6 h) may lead to degradation of protoplast quality, while shorter incubations (< 3 h) prevent complete cell wall digestion and will lower protoplast yields.*

3.4 Add 30 mL W5 Solution and shake gently by hand to stop digestion. Filter the sample through 40 µm mesh by gravity to release protoplasts into 50 ml tubes. Rinse the plant tissue retained by the mesh with an additional 60 mL W5 Solution. *NOTE – gently agitate the plant tissue remaining in the mesh during rinsing to improve protoplast yield.*

3.5 Gently pellet the protoplasts by centrifuging all flow-through from step 3.4 (120 mL total) at 250 x g for 3 min at RT. Carefully aspirate or decant the supernatant. *NOTE – at this stage, decanting the supernatant is easier. Tubes can then be inverted onto paper tissue for 1 min to remove as much supernatant as possible.*

3.6 Wash the pellet by gently resuspending in 10 mL W5 buffer, then centrifuging at 250 x g for 3 min at RT. Carefully aspirate or decant the supernatant and resuspend the pellet in 2 mL MMG Solution. *NOTE – after resuspending the pellet, if the solution is not homogenous, filter again through 40 µm mesh to remove any clumps.*

4. Transformation of protoplasts

4.1 Combine 5 µg pDNA (in a volume of 10 µL, diluted with ddH_2O) and 60 µL protoplast suspension in a 1.5 mL microcentrifuge tube. Include a negative control sample (60 µL protoplasts, 10 µL ddH_2O, no pDNA).

4.2 Add 70 µL PEG Solution and mix gently by inverting/rotating the tube. Incubate in the dark at RT for 25 min to allow transformation to occur.

4.3 Add 280 µL W5 Solution and mix gently by inverting to terminate the transformation process. Centrifuge at 250 x g for 3 min at RT. Carefully aspirate the supernatant, and resuspend the pellets in 500 µL WI Solution. *NOTE – remove as much supernatant as possible at this stage. Resuspend by inverting tubes, rather than by pipetting.*

4.4 Dispense 125 µL aliquots into a 96-well microplate. Incubate at RT on the lab bench for 16 h, during which time the transgenic protein(s) will accumulate. *NOTE – it is essential to cover the microplate with a lid during this incubation.*

5. Confocal imaging of transformed protoplasts (optional)

5.1 If the protoplasts have settled to the bottom of the microplate wells, resuspend with very gentle pipetting. Pipette a small drop of transformed protoplasts onto a microscope slide inside a well constructed from observation gel, place a cover slip over the sample, then press down gently to seal the cover slip onto the observation gel.

5.2 Ensure excitation lasers for the confocal microscope are switched on and fully warmed-up. Visualise fluorescent signal by exciting with the appropriate laser and collecting an appropriate emission bandwidth for the fluorescent tag used.

5.3 To confirm localisation, include a sample treated with a specific stain for the expected site of localisation (chlorophyll autofluorescence for chloroplasts), or an alternative positive control (e.g. a fluorescent protein fused to a protein or transit peptide conferring known localisation).
