# Supplementary material for: A high‐throughput transient expression system for rice
Source: Plant Cell Environ. 2019 Apr 2;42(7):2057–64. doi: 10.1111/pce.13542 (PMC6618034; doi:10.1111/pce.13542)
Supplement: Supplementary file 3 — Table S1. Sequencing primers used to verify correct assembly of plasmids. Primer binding positions can be seen on the plasmid maps in Supplementary Figure S1. Supplementary Figure S1(a). Map of Plasmid 1. This plasmid was used for the determination of transformation efficiency when altering pDNA amount, protoplast volume, and final PEG concentration (data in Figure 3b‐d). Primer sequences are given in Supplementary Table S1. Supplementary Figure S1(b). Map of Plasmid 2. This plasmid was used for localisation studies of RecA‐CcmK2‐YFP (data in Figure 4a). Primer sequences are given in Supplementary Table S1. Supplementary Figure S1(c). Map of Plasmid 3. This plasmid was used for localisation studies of RecA‐CcmL‐YFP (data in Figure 4a). Primer sequences are given in Supplementary Table S1. Supplementary Figure S1(d). Map of Plasmid 4. This plasmid was used for localisation studies of RecA‐CcmO‐YFP (data in Figure 4a). Primer sequences are given in Supplementary Table S1. Supplementary Figure S1(e). Map of Plasmid 5. This plasmid was used for localisation studies of RecA‐CcmP‐YFP (data in Figure 4a). Primer sequences are given in Supplementary Table S1. Supplementary Figure S1(f). Map of Plasmid 6. This plasmid was used for localisation studies of RecA‐RbcL‐YFP (data in Figure 4b). Primer sequences are given in Supplementary Table S1. Supplementary Figure S1(g). Map of Plasmid 7. This plasmid was used for localisation studies of RecA‐RbcS‐YFP (data in Figure 4b). Primer sequences are given in Supplementary Table S1. Supplementary Figure S1(h). Map of Plasmid 8. This plasmid was used for localisation studies of RecA‐CcaA‐YFP (data in Figure 4b). Primer sequences are given in Supplementary Table S1. Supplementary Figure S1(i). Map of Plasmid 9. This plasmid was used for localisation studies of RecA‐CcmM58‐YFP (data in Figure 4b). Primer sequences are given in Supplementary Table S1. Supplementary Figure S1(j). Map of Plasmid 10. This plasmid was used for localisation st [file PCE-42-2057-s003.pdf]

Supplementary Table S1. Sequencing primers used to verify correct assembly of plasmids. Primer binding positions can be seen on the plasmid maps in Supplementary Figure S1.

Primer Name	Sequence (5' to 3')
Primer 1	GTGGCAGGATATATTGTGGTG
Primer 2	GCCAATATATCCTGTCAAACACTG
Primer 3	GAAGTGCTGTCGCACCACATC
Primer 4	GAAGTGAAGTTCGAGGGCGA
Primer 5	GATGCAGCTGTCATTGCCATC
Primer 6	CAAGGCACGGTTTCTAATGTG
Primer 7	CTGGAGAACTTGTCGGGGAGA
Primer 8	CAAGCTGGAGGGCGACAAGG
Primer 9	GACAACATTAAGCAGTGTCAGACC
Primer 10	GAACCTCAAGACCTACCCAATC
Primer 11	CAATGGCTCTTCATCGTCCG
Primer 12	GTGGTGTAAACAAATTGACGC
Primer 13	GGATAAACCTTTTCACGCCC
Primer 14	CTCTCCTTCGCCTTCTATTTC

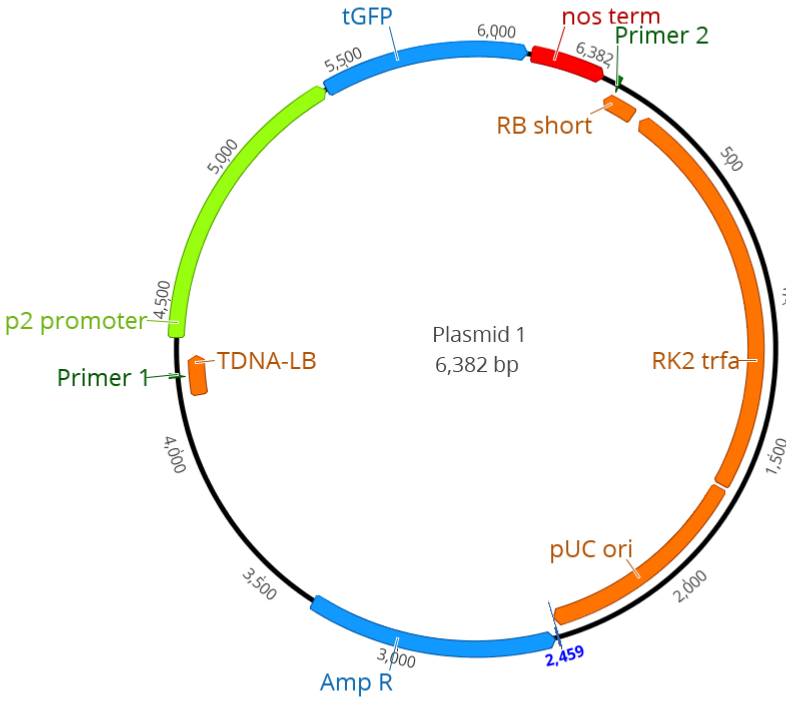

Supplementary Figure S1(a). Map of Plasmid 1. This plasmid was used for the determination of transformation efficiency when altering pDNA amount, protoplast volume, and final PEG concentration (data in Figure 3b-d). Primer sequences are given in Supplementary Table S1.

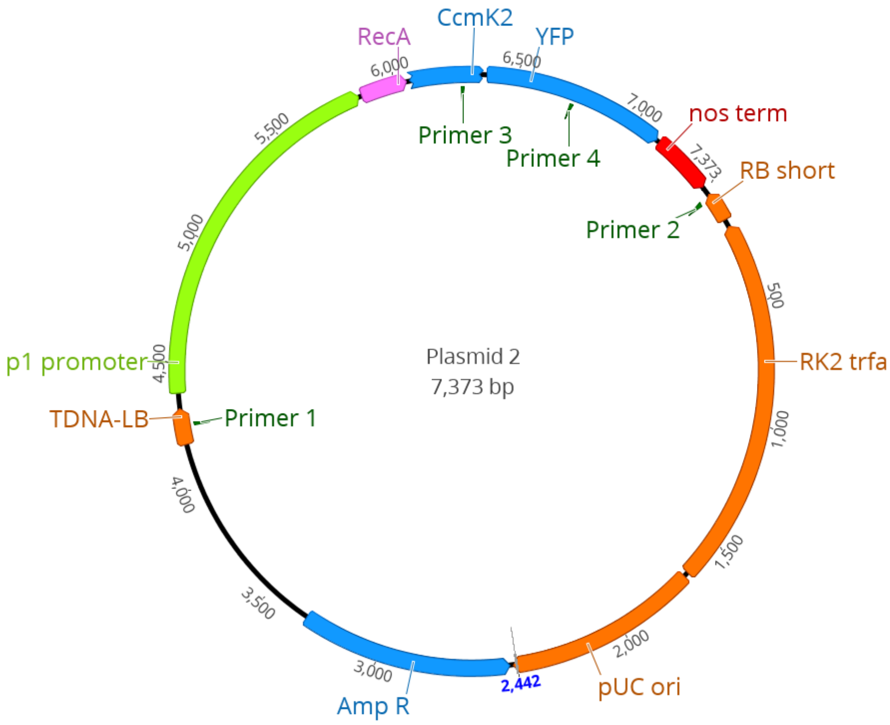

Supplementary Figure S1(b). Map of Plasmid 2. This plasmid was used for localisation studies of RecA-CcmK2-YFP (data in Figure 4a). Primer sequences are given in Supplementary Table S1.

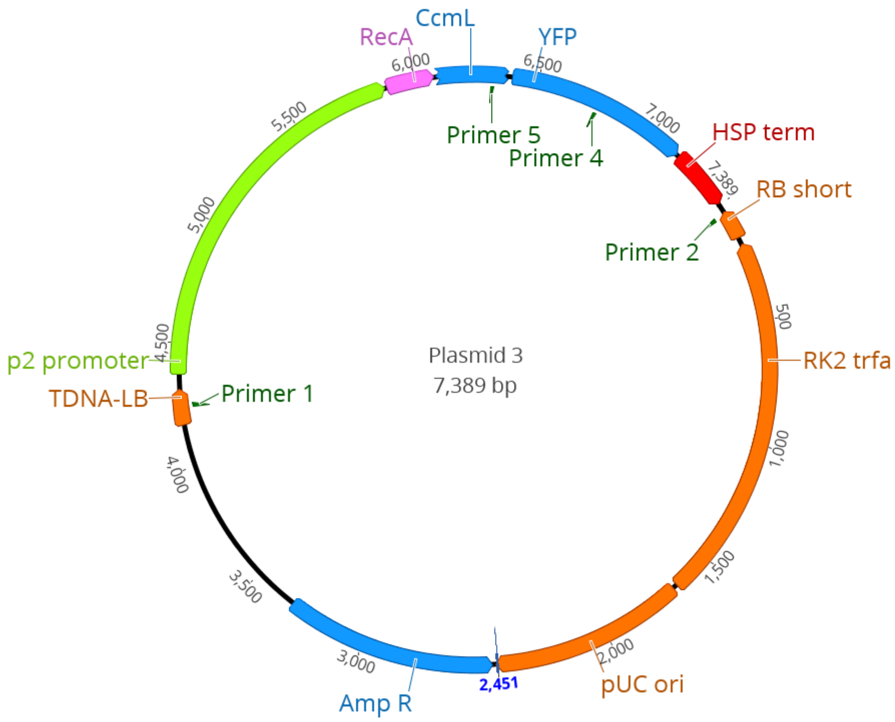

Supplementary Figure S1(c). Map of Plasmid 3. This plasmid was used for localisation studies of RecA-CcmL-YFP (data in Figure 4a). Primer sequences are given in Supplementary Table S1.

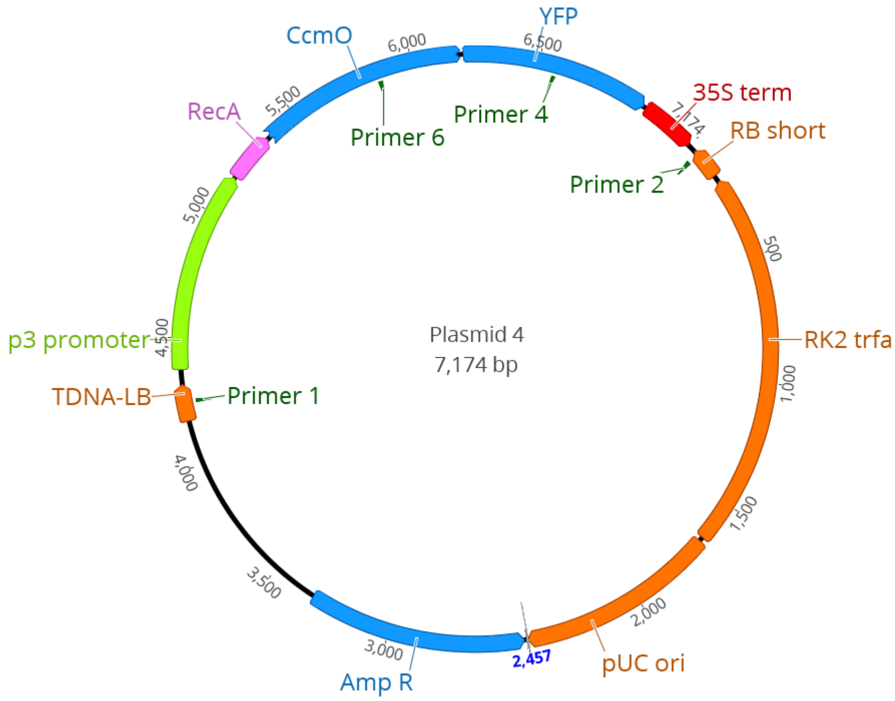

Supplementary Figure S1(d). Map of Plasmid 4. This plasmid was used for localisation studies of RecA-CcmO-YFP (data in Figure 4a). Primer sequences are given in Supplementary Table S1.

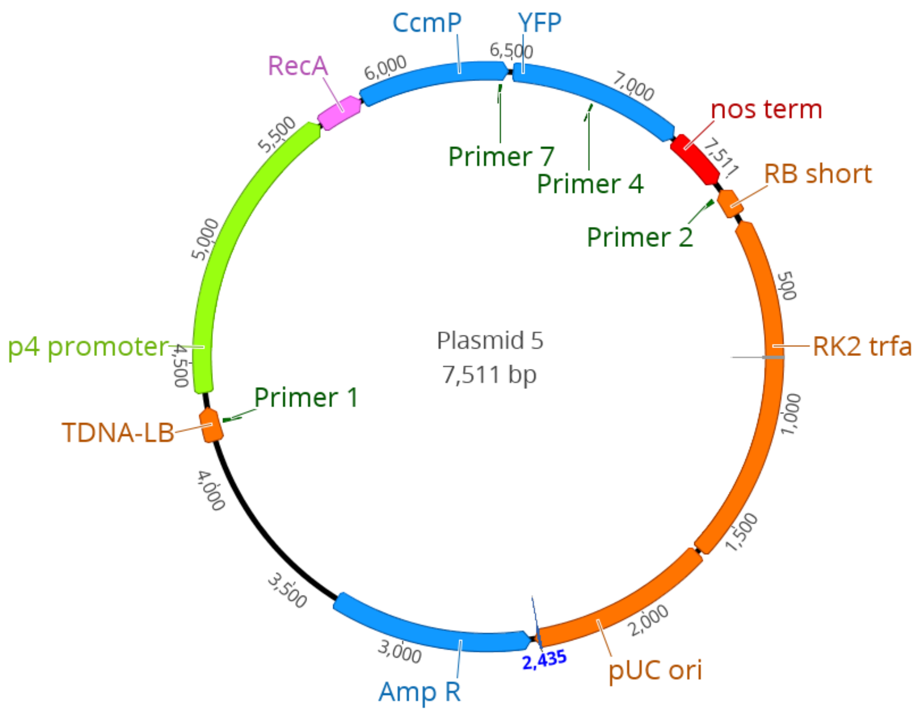

Supplementary Figure S1(e). Map of Plasmid 5. This plasmid was used for localisation studies of RecA-CcmP-YFP (data in Figure 4a). Primer sequences are given in Supplementary Table S1.

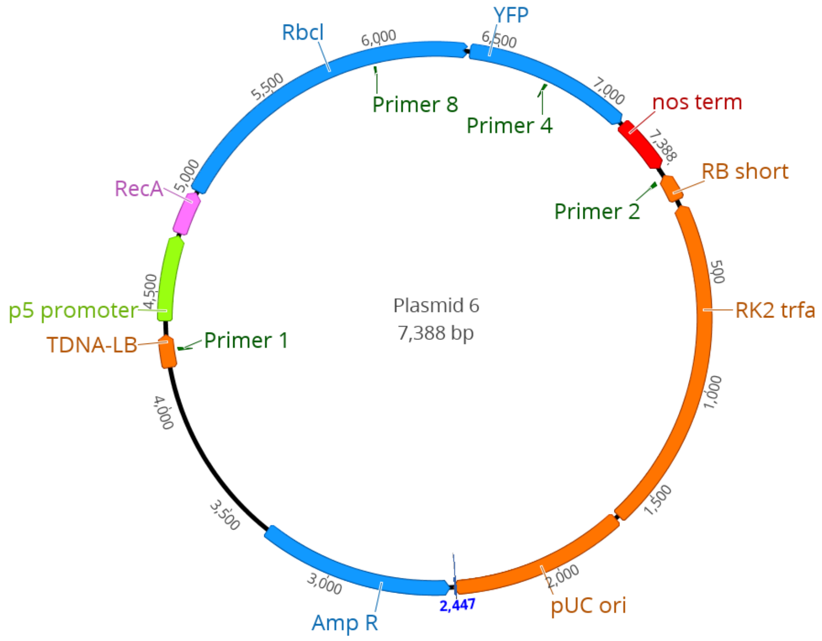

Supplementary Figure S1(f). Map of Plasmid 6. This plasmid was used for localisation studies of RecA-RbcL-YFP (data in Figure 4b). Primer sequences are given in Supplementary Table S1.

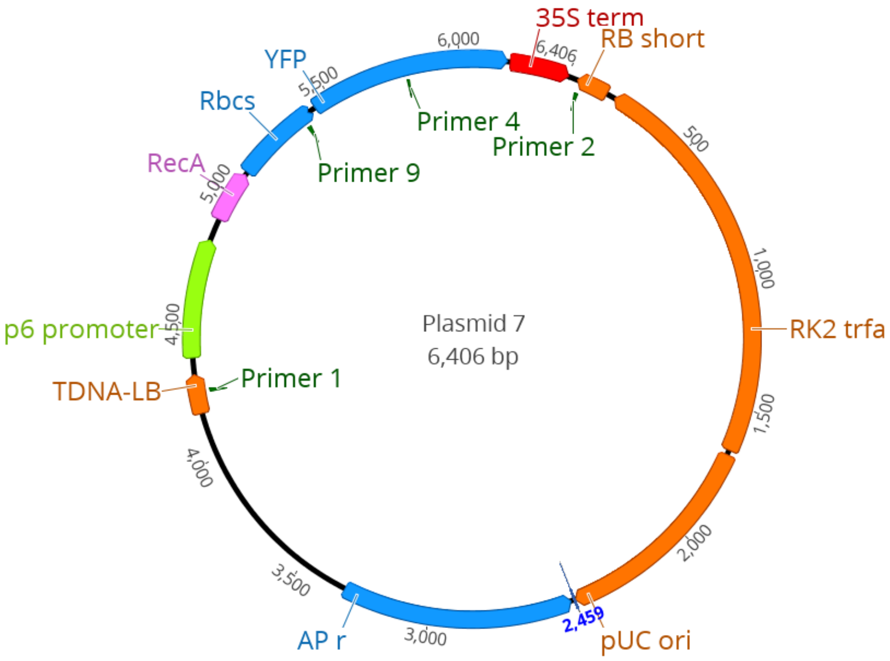

Supplementary Figure S1(g). Map of Plasmid 7. This plasmid was used for localisation studies of RecA-RbcS-YFP (data in Figure 4b). Primer sequences are given in Supplementary Table S1.

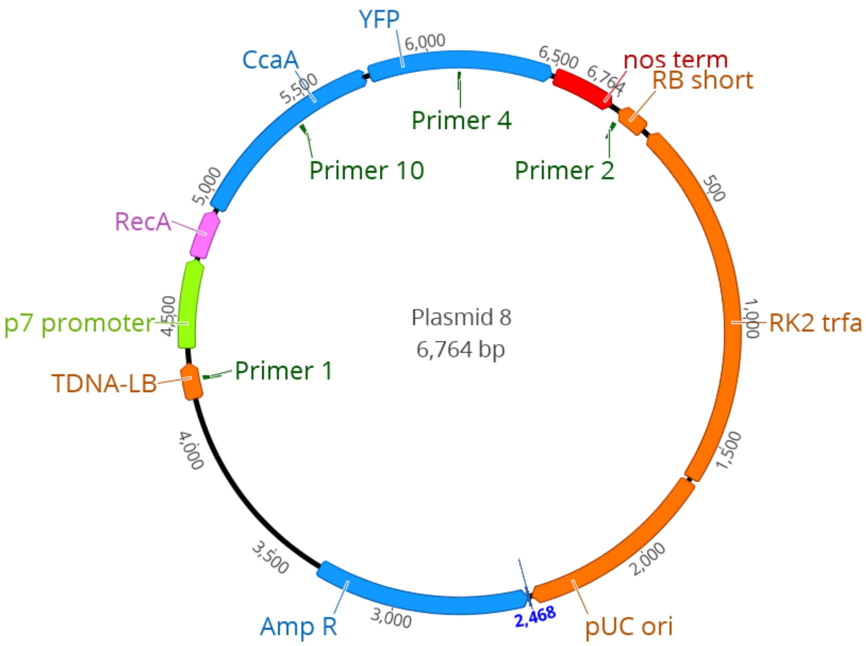

Supplementary Figure S1(h). Map of Plasmid 8. This plasmid was used for localisation studies of RecA-CcaA-YFP (data in Figure 4b). Primer sequences are given in Supplementary Table S1.

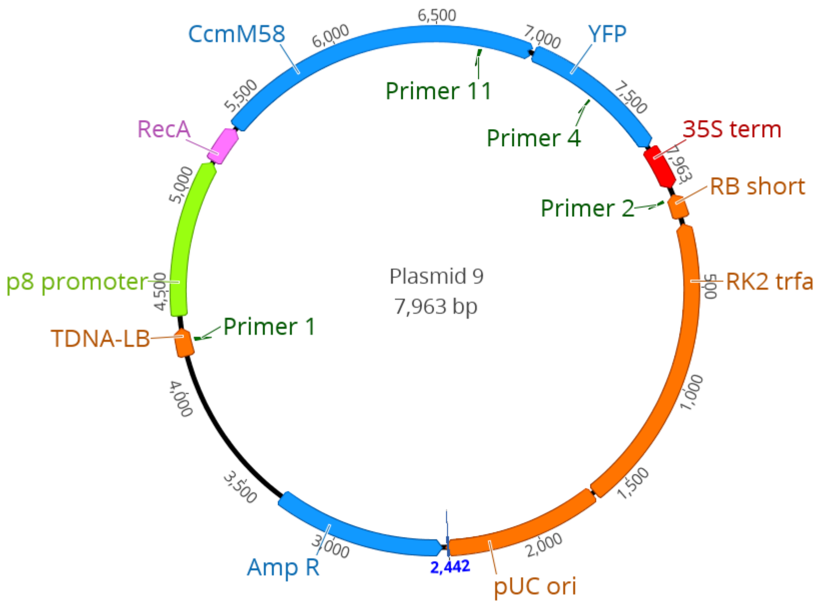

Supplementary Figure S1(i). Map of Plasmid 9. This plasmid was used for localisation studies of RecA-CcmM58-YFP (data in Figure 4b). Primer sequences are given in Supplementary Table S1.

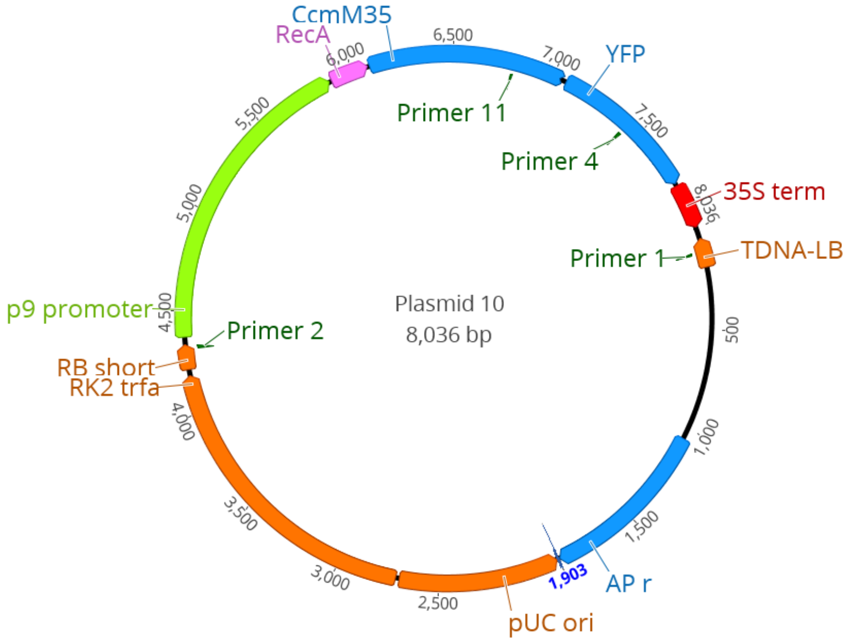

Supplementary Figure S1(j). Map of Plasmid 10. This plasmid was used for localisation studies of RecA-CcmM35-YFP (data in Figure 4b). Primer sequences are given in Supplementary Table S1.

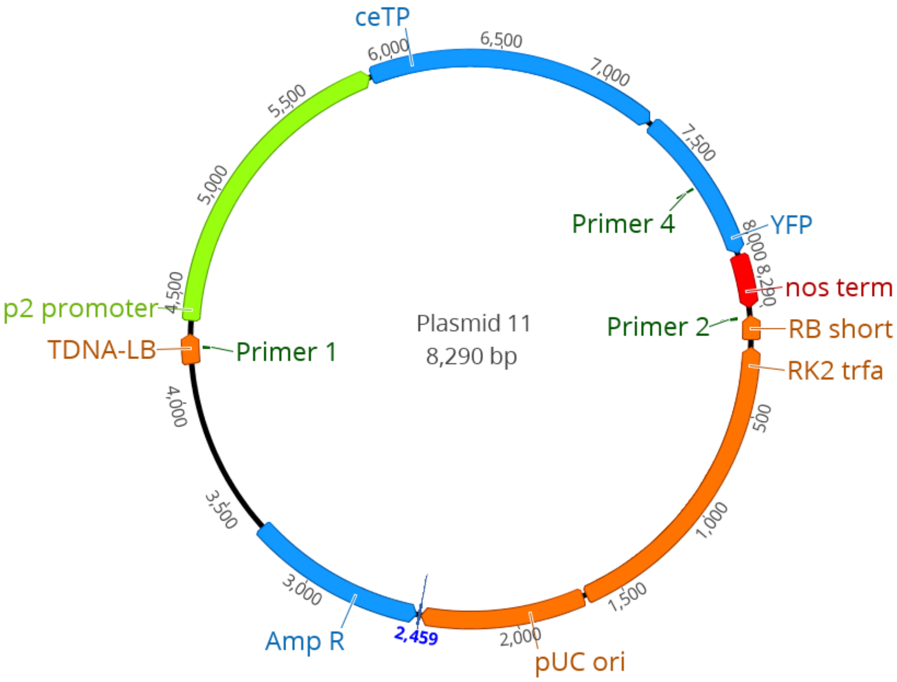

Supplementary Figure S1(k). Map of Plasmid 11. This plasmid was used determine localisation of a fluorescent protein (YFP) directed by a chloroplast envelope transit peptide (ceTP, data in Figure 4c). Primer sequences are given in Supplementary Table S1.

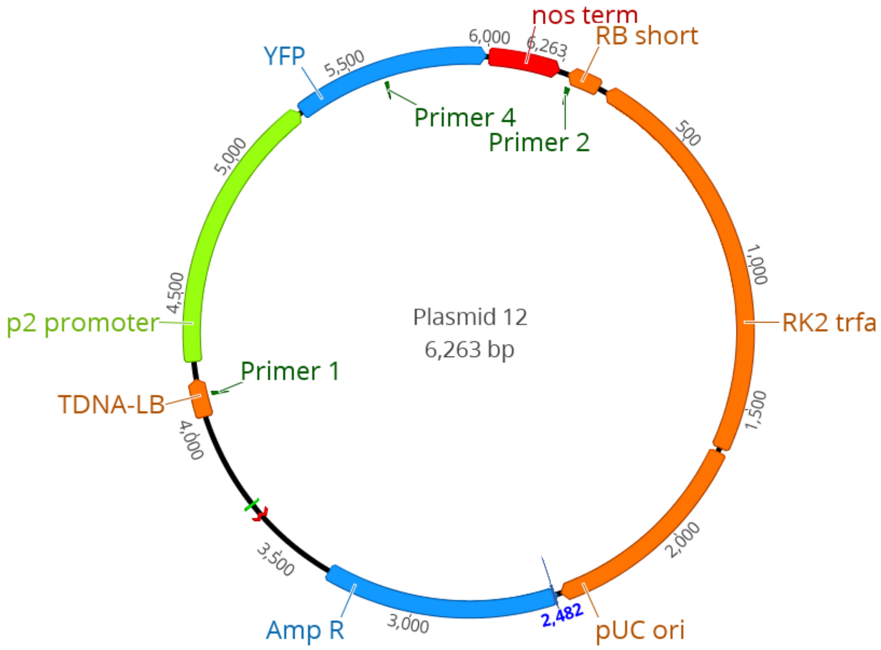

Supplementary Figure S1(l). Map of Plasmid 12. This plasmid was used determine localisation of a fluorescent protein (YFP) in the absence of a transit peptide (data in Figure 4c). Primer sequences are given in Supplementary Table S1.

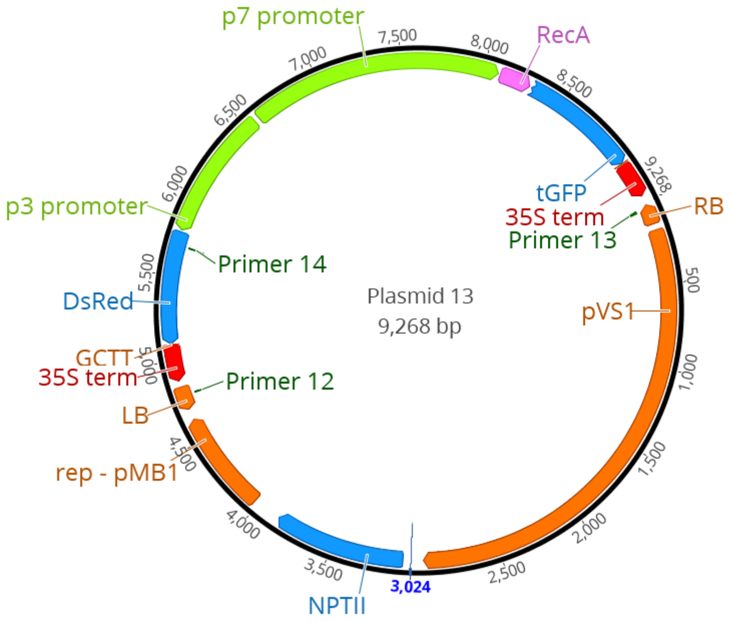

Supplementary Figure S1(m). Map of Plasmid 13. This plasmid was used determine localisation of two fluorescent proteins: DsRed in the absence of a transit peptide, and tGFP directed by a chloroplast stroma transit peptide (RecA, data in Figure 4c). Primer sequences are given in Supplementary Table S1.

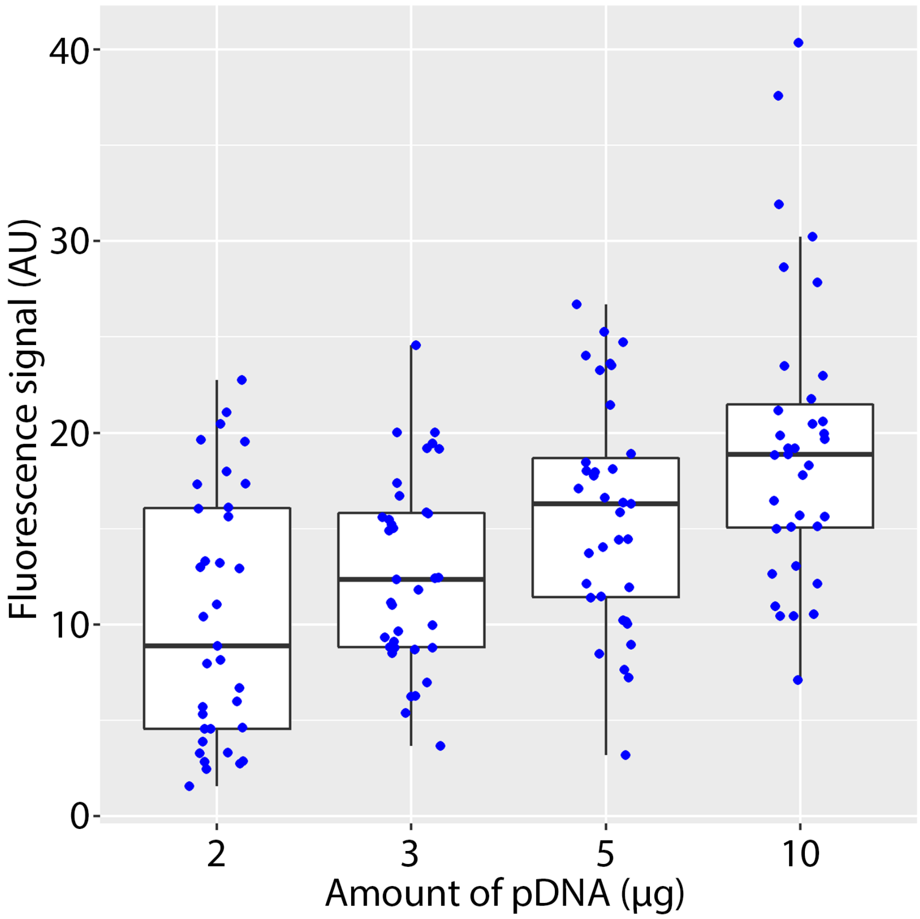

Supplementary Figure S2. The effect of pDNA amount on tGFP signal intensity. Box-whisker plots to show the distribution of signal intensities when different amounts of plasmid DNA (pDNA) are used to transform protoplasts. The boxes represent the lower quartile, median, and upper quartile. Whiskers extend to the minimum and maximum data values for each sample (excluding outliers). Consistent confocal microscope settings (laser power, gain) were used to image all samples. All individual data points are shown in blue (n = 35).

Additional files can also be downloaded separately:

Supplementary Protocol S1. A detailed protocol for rice protoplast isolation and transformation, including reagents and materials required.

Supplementary Video S1. A time-lapse video to accompany Supplementary Protocol S1, with numbered steps matching the steps in the written protocol.
